# Inhibitory Effects of Quercetin and Its Human and Microbial Metabolites on Xanthine Oxidase Enzyme

**DOI:** 10.3390/ijms20112681

**Published:** 2019-05-31

**Authors:** Violetta Mohos, Attila Pánovics, Eszter Fliszár-Nyúl, Gabriella Schilli, Csaba Hetényi, Přemysl Mladěnka, Paul W. Needs, Paul A. Kroon, Gábor Pethő, Miklós Poór

**Affiliations:** 1 Department of Pharmacology, University of Pécs, Faculty of Pharmacy, Szigeti út 12, H-7624 Pécs, Hungary; mohos.violetta@gytk.pte.hu (V.M.); attilapanovics@gmail.com (A.P.); eszter.nyul@aok.pte.hu (E.F.-N.); gabor.petho@aok.pte.hu (G.P.); 2 János Szentágothai Research Center, University of Pécs, Ifjúság útja 20, H-7624 Pécs, Hungary; 3 Department of Pharmacology and Pharmacotherapy, University of Pécs, Medical School, Szigeti út 12, H-7624 Pécs, Hungary; scgpecs@gmail.com (G.S.); csabahete@yahoo.com (C.H.); 4 Department of Pharmacology and Toxicology, Faculty of Pharmacy in Hradec Králové, Charles University, Heyrovského 1203, 500 05 Hradec Králové, Czech Republic; mladenkap@faf.cuni.cz; 5 Quadram Institute Bioscience, Norwich Research Park, Norwich NR4 7UA, UK; paul.needs@quadram.ac.uk (P.W.N.); paul.kroon@quadram.ac.uk (P.A.K.)

**Keywords:** xanthine oxidase, quercetin metabolites, 6-mercaptopurine, xanthine, enzyme inhibition, pharmacokinetic interactions

## Abstract

Quercetin is an abundant flavonoid in nature and is used in several dietary supplements. Although quercetin is extensively metabolized by human enzymes and the colonic microflora, we have only few data regarding the pharmacokinetic interactions of its metabolites. Therefore, we investigated the interaction of human and microbial metabolites of quercetin with the xanthine oxidase enzyme. Inhibitory effects of five conjugates and 23 microbial metabolites were examined with 6-mercaptopurine and xanthine substrates (both at 5 μM), employing allopurinol as a positive control. Quercetin-3′-sulfate, isorhamnetin, tamarixetin, and pyrogallol proved to be strong inhibitors of xanthine oxidase. Sulfate and methyl conjugates were similarly strong inhibitors of both 6-mercaptopurine and xanthine oxidations (IC_50_ = 0.2–0.7 μM); however, pyrogallol inhibited xanthine oxidation (IC_50_ = 1.8 μM) with higher potency vs. 6-MP oxidation (IC_50_ = 10.1 μM). Sulfate and methyl conjugates were approximately ten-fold stronger inhibitors (IC_50_ = 0.2–0.6 μM) of 6-mercaptopurine oxidation than allopurinol (IC_50_ = 7.0 μM), and induced more potent inhibition compared to quercetin (IC_50_ = 1.4 μM). These observations highlight that some quercetin metabolites can exert similar or even a stronger inhibitory effect on xanthine oxidase than the parent compound, which may lead to the development of quercetin–drug interactions (e.g., with 6-mercaptopurin or azathioprine).

## 1. Introduction

**Figure 1:**
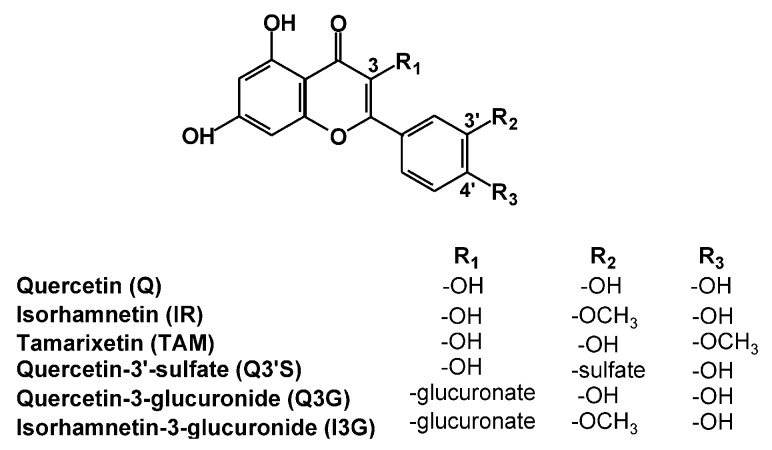
Chemical structures of quercetin and its conjugated metabolites with preserved flavonoid structure.

Quercetin (Q) is a flavonoid; it is found in many commonly consumed foods and beverages including apples, onions, tea, and red wine [1]. It is included in dietary supplements in high doses (from several hundreds to thousands of milligrams). These supplements are advertised and freely available through the Internet for several purported uses. The oral bioavailability of Q is low, due to its poor aqueous solubility and significant presystemic elimination [2,3]. As a result of the metabolism, Q is known to form methyl, sulfate, and glucuronic acid conjugates, such as 3′-O-methylquercetin (isorhamnetin, IR), 4′-O-methylquercetin (tamarixetin, TAM), quercetin-3′-sulfate (Q3′S), quercetin-3-glucuronide (Q3G), and isorhamnetin-3-glucuronide (I3G) (Figure 1) [3,4,5,6]. Q3′S, Q3G, and I3G are the main circulating metabolites of Q with preserved flavonoid core in humans [7]. Even after high dietary intake of Q, total plasma Q (the parent compound and its metabolites) commonly only reaches nanomolar concentrations [3,6]. Micromolar total plasma Q concentrations were detected only after the administration of dietary supplements with exceedingly high Q content (e.g., 1000 mg/day) [8]. Furthermore, Q is biotransformed by the colonic microflora through degradation of the flavonoid ring(s) into smaller products. These can be classified in four major groups: hydroxybenzoic, hydroxyacetic, and hydroxycinnamic acids, and hydroxybenzenes [6,9,10,11].

Xanthine oxidase (XO) is a molybdenum-containing enzyme playing an important role in purine catabolism [12]. The antitumor drug 6-mercaptopurine (6-MP) is also biotransformed by XO, yielding pharmacologically inactive 6-thiouric acid (6-TU) [13]. Allopurinol (APU) is a well-known XO inhibitor used in the treatment of hyperuricemia or gout [12]. Besides the inhibition of uric acid formation, the inhibition of XO may also be beneficial by decreasing superoxide radicals under some pathological conditions [14,15]. However, the simultaneous administration of APU and 6-MP slows elimination of the latter compound, which may cause toxic effects, i.e., severe bone marrow depression [16].

Based on previous studies, Q is a strong competitive inhibitor of XO [17,18,19], while other authors suggest the involvement of noncompetitive mechanisms as well [20,21]. The effect of Q on the XO-catalyzed oxidation of xanthine has been described; however, the influence of Q on 6-MP oxidation has not been reported. Furthermore, as mentioned above, Q undergoes significant presystemic elimination; therefore, its metabolites reach higher concentrations in blood (and likely in tissues) than the parent compound, Q. Considering the fact that some Q metabolites produce similar or even stronger interaction with some proteins than Q itself [22,23], it is reasonable to hypothesize that some Q metabolites may influence the XO-catalyzed xanthine and/or 6-MP oxidation. Since the inhibition of 6-MP elimination may have serious adverse consequences, we aimed to investigate the interactions of Q as well as its human and microbial metabolites (including also other flavonoid microbial metabolites) with XO enzyme employing xanthine and 6-MP substrates as well as APU and oxipurinol (the active metabolite of APU) as positive controls.

## 2. Results

### 2.1. Inhibitory Effects of Q and Its Human Metabolites on XO-Catalyzed 6-MP Oxidation

**Figure 2:**
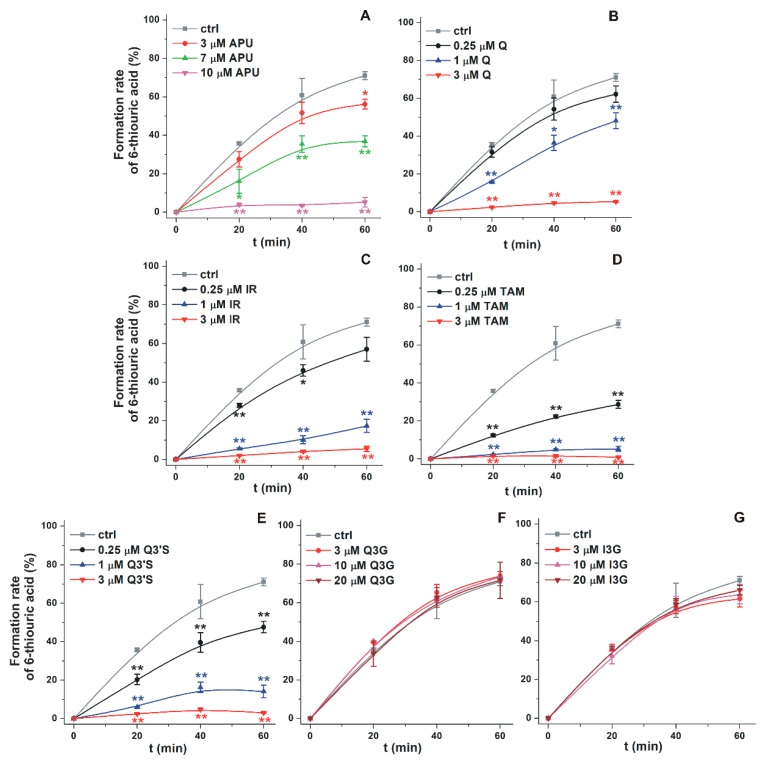
The time course of XO-catalyzed formation of 6-thiouric acid in the presence of increasing concentrations of APU, Q, and conjugated Q metabolites (see further details in the Materials and Methods). Graphs depict % conversion of 6-MP to 6-TU in the absence and presence of increasing concentrations of allopurinol (APU; **A**), quercetin (Q; **B**), isorhamnetin (IR; **C**), tamarixetin (TAM; **D**), quercetin-3′-sulfate (Q3′S; **E**), quercetin-3-glucuronide (Q3G; **F**), and isorhamnetin-3-glucuronide (I3G; **G**) (* *p* < 0.05; ** *p* < 0.01).

First, the effects of Q and its conjugated metabolites on 6-MP oxidation were examined. Figure 2 demonstrates the time-dependence of 6-TU formation in the absence and presence of Q metabolites, where % conversion of 6-MP to 6-TU is expressed. Even relatively high concentrations (20 μM = four-fold excess vs. the substrate) of the glucuronide conjugates (Q3G and I3G) did not inhibit the formation of 6-TU. However, Q, as well as its methylated (IR and TAM) and sulfated (Q3′S) metabolites, proved to be strong inhibitors, even stronger than the positive control APU (Figure 2). Q, Q3′S, IR, and TAM at 3 μM concentration almost completely inhibited 6-TU formation, while the same concentration of APU caused only a slight effect. Furthermore, Q showed only weak inhibitory action at 0.25 μM concentration, while at this concentrations Q3′S and TAM significantly decreased the metabolite formation throughout incubation.

**Figure 3:**
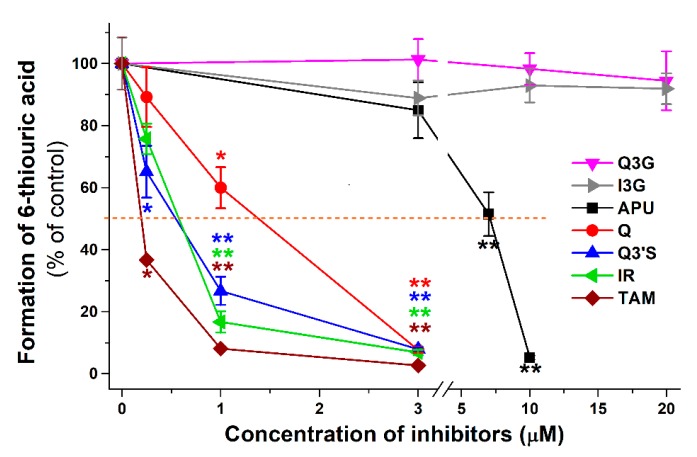
Inhibitory effects of Q and its conjugated metabolites on XO-catalyzed oxidation of 6-MP (5 μM) after 40 min incubation, in the presence of increasing concentrations of allopurinol (APU), quercetin (Q), isorhamnetin (IR), tamarixetin (TAM), quercetin-3′-sulfate (Q3′S), quercetin-3-glucuronide (Q3G), and isorhamnetin-3-glucuronide (I3G). The 50% inhibition of 6-thiouric acid formation (IC_50_) is marked with dashed line (* *p* < 0.05; ** *p* < 0.01).

**Figure 4:**
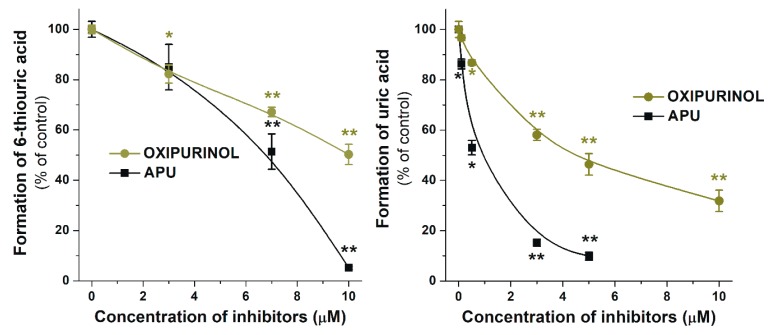
Inhibitory effects of oxipurinol and allopurinol (APU) on XO-catalyzed oxidation of 6-MP and xanthine after 40 and 8 min incubations, respectively. * *p* < 0.05; ** *p* < 0.01).

Figure 3 demonstrates the concentration-dependent inhibitory effect of APU, Q, and conjugated Q metabolites on the formation of 6-TU. These experiments also highlight the strong inhibitory effects of TAM, Q3′S, IR, and Q on 6-MP oxidation. Based on Figure 3, the IC_50_ values (i.e., the concentrations causing 50% decrease in metabolite formation) of Q and its metabolites were determined. Q (IC_50_ = 1.4 μM) was a five-fold stronger inhibitor than APU (IC_50_ = 7.0 μM), while the IC_50_ values of Q3′S, IR, and TAM were in the 0.2–0.5 μM range and showed approximately ten-fold stronger inhibition of XO-catalyzed 6-MP oxidation than the positive control APU (Table 1). Furthermore, these conjugates were two- to seven-fold stronger inhibitors of 6-TU formation than the parent compound Q. The IC_50_ values of Q, Q3′S, IR, and TAM (0.2–1.4 μM) were much lower than the substrate concentration (5 μM). As the active metabolite of APU, the inhibitory effect of oxipurinol was also tested. Oxipurinol (IC_50_ = 10 μM) was a significant but weaker inhibitor of XO-catalyzed oxidation of 6-MP than APU (Figure 4, left).

**Figure 5:**
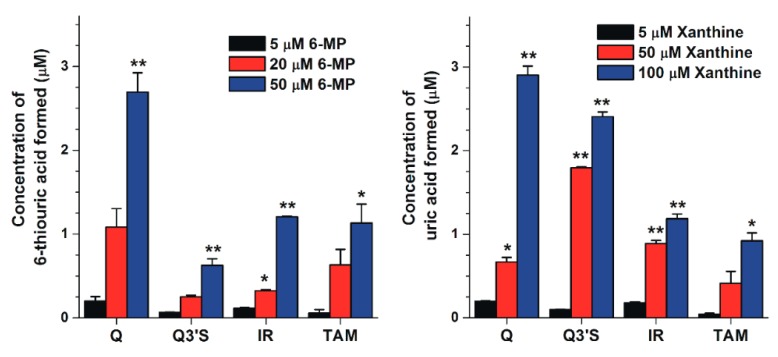
Concentrations of 6-thiouric acid (**left**) and uric acid (**right**) formed in the presence of 3 and 10 μM flavonoid concentrations, respectively (substrate concentrations are indicated in the figure; * *p* < 0.05, ** *p* < 0.01).

In another experiment, the reversibility of the inhibition was tested in the presence of 3 μM flavonoids (i.e., Q, Q3′S, IR, or TAM): XO was preincubated with the flavonoids for 15 min, after which, the substrate (6-MP) was added at increasing concentrations (5–50 μM) and the product formation was determined after a subsequent 40 min incubation. The flavonoids at 3 μM concentrations almost completely abolished the metabolite formation in the presence of 5 μM substrate (see in Figure 3); however, at higher concentrations of 6-MP, the metabolite formation increased markedly in a concentration-dependent fashion (Figure 5).

### 2.2. Inhibitory Effects of Q and Its Human Metabolites on XO-Catalyzed Xanthine Oxidation

**Figure 6:**
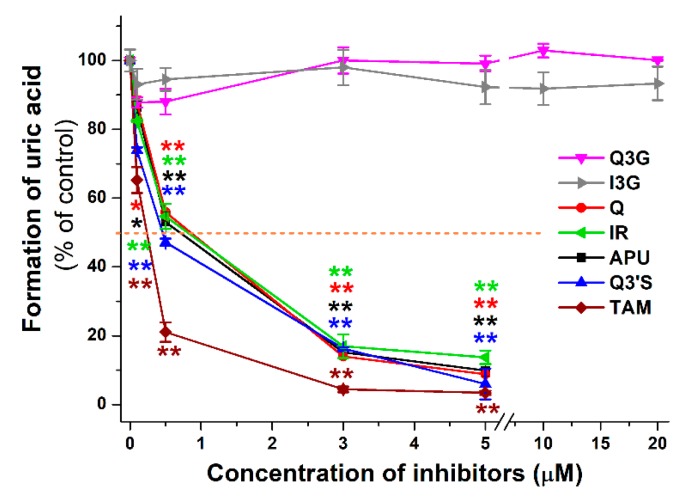
Inhibitory effects of Q and its conjugated metabolites on XO-catalyzed oxidation of xanthine (5 μM) after 8 min incubations, in the presence of increasing concentrations of allopurinol (APU), quercetin (Q), isorhamnetin (IR), tamarixetin (TAM), quercetin-3′-sulfate (Q3′S), quercetin-3-glucuronide (Q3G), and isorhamnetin-3-glucuronide (I3G). The 50% inhibition of uric acid formation (IC_50_) is marked with dashed line (* *p* < 0.05, ** *p* < 0.01).

The effects of Q and its conjugated metabolites on xanthine oxidation were also tested (Figure S1). Figure 6 demonstrates the concentration-dependent inhibitory effect of flavonoids on XO-catalyzed uric acid formation. Similar to the previous assay (see in Figure 3), glucuronide conjugates (Q3G and I3G) did not inhibit the XO activity even at four-fold concentration compared to the substrate. However, Q, as well as its methyl and sulfate conjugates, exerted a strong inhibitory effect on XO-catalyzed uric acid formation. Q, Q3′S, and IR inhibited xanthine oxidation to a similar extent as the positive control APU, whereas TAM was a stronger inhibitor compared to these compounds. As Table 1 demonstrates, IC_50_ values of APU, Q, Q3′S, IR, and TAM are in the same range (0.20–0.80 μM). These data highlight that Q as well as its methyl and sulfate conjugates are similarly strong inhibitors of XO-catalyzed xanthine oxidation than APU, producing a 50% decrease in metabolite formation at approximately 1/10th of the substrate concentration. The effect of oxipurinol was also tested; however, it induced significantly weaker effect (IC_50_ = 4.5 μM) on uric acid formation than APU (0.6 μM; Figure 4, right).

We examined the reversibility of the inhibition. XO was preincubated with 10 μM flavonoid (Q, Q3′S, IR, or TAM) for 15 min, after which the substrate (xanthine) was added at increasing concentrations (5–100 μM) and uric acid formation was determined after a subsequent 8 min incubation. These flavonoids at 10 μM concentrations almost completely abolished urate formation from 5 μM xanthine (see in Figure 6); however, xanthine strongly enhanced the metabolite formation in a concentration-dependent fashion (Figure 5).

### 2.3. Inhibitory Effects of Q, Q3′S, APU, and Oxipurinol on XO-Catalyzed Hypoxanthine Oxidation

**Figure 7:**
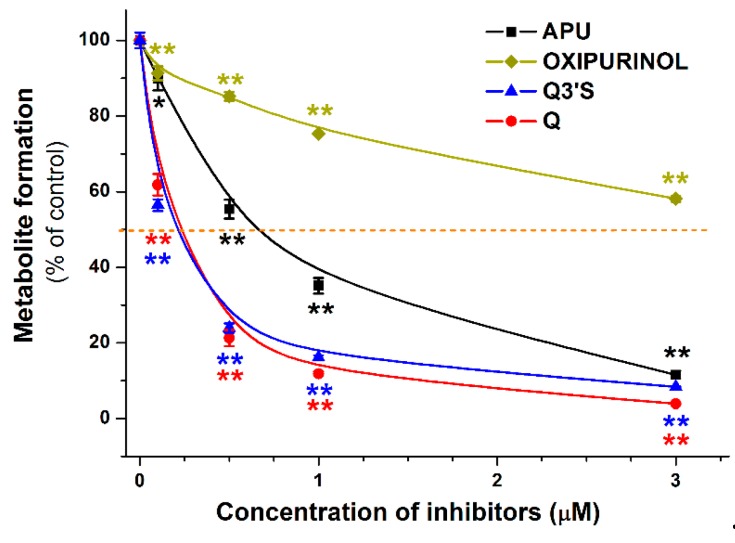
Inhibitory effects of quercetin (Q), quercetin-3′-sulfate (Q3′S), allopurinol (APU), and oxipurinol on XO-catalyzed oxidation of hypoxanthine (5 μM) after 4 min incubations. The 50% inhibition of metabolite (xanthine + uric acid) formation (IC_50_) is marked with dashed line (* *p* < 0.05, ** *p* < 0.01).

Because xanthine is conventionally applied to examine XO activity, the effects of flavonoids on 6-MP oxidation were compared with xanthine oxidation. It is important to note that 6-MP forms 6-TU through two oxidation steps, while uric acid formation from xanthine is a one-step reaction. Therefore, we tested the effects of Q, Q3′S, APU, and oxipurinol on XO-catalyzed hypoxanthine oxidation, which is a two-step reaction (hypoxanthine → xanthine → uric acid), similar to the 6-MP oxidation (6-MP → 6-thipxanthine → 6-TU). During the hypoxanthine assay (see details in Section 4.4), as significant concentrations of both xanthine and uric acid appeared in incubates, the quantities of formed xanthine and uric acid were expressed as the sum of both. As Figure 7 demonstrates, oxipurinol induced weaker inhibition of hypoxanthine oxidation than APU, in accordance with xanthine and 6-MP oxidation (Figure 4). However, Q and Q3′S proved to be approximately three-fold stronger inhibitors of hypoxanthine oxidation than APU (Table 2). The IC_50_ values of APU, Q, and Q3′S for hypoxanthine oxidation were in the same range as for xanthine oxidation (Table 1).

### 2.4. Inhibitory Effects of the Microbial Metabolites on XO-Catalyzed 6-MP and Xanthine Oxidation

**Figure 8:**
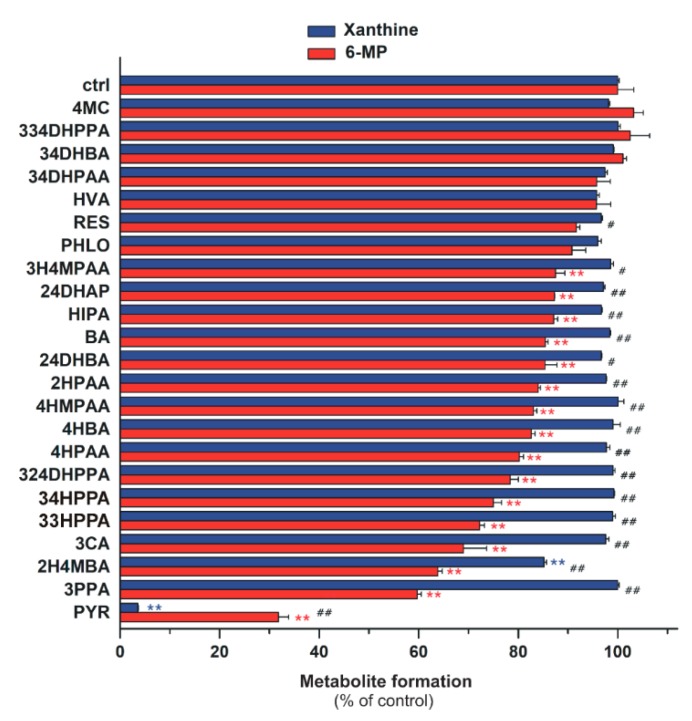
Effects of microbial flavonoid metabolites (each 20 μM) on XO-catalyzed xanthine (5 μM) and 6-MP (5 μM) oxidation. The hash signs indicate significant differences (# *p* < 0.05, ## *p* < 0.01) between xanthine and 6-MP oxidation. Whereas the asterisks mark significant decreases (* *p* < 0.05, ** *p* < 0.01) in xanthine or 6-MP oxidation, as compared to that of control.

**Figure 9:**
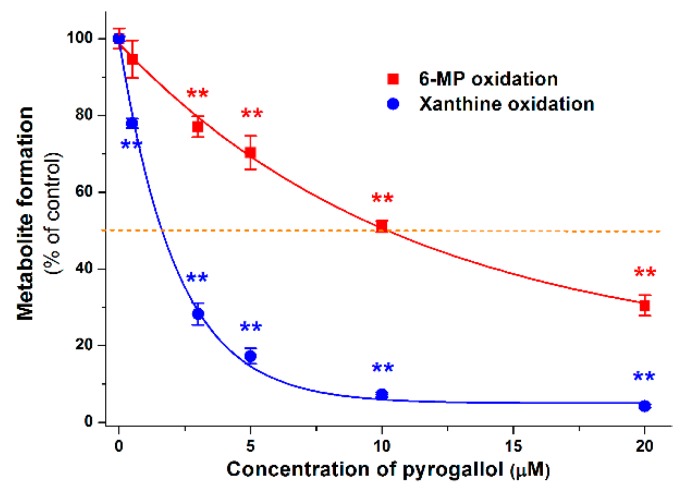
Inhibitory effect of PYR on 6-MP and xanthine oxidation. The 50% inhibition of metabolite formation (IC_50_) is marked with dashed line (* *p* < 0.05; ** *p* < 0.01).

Since several absorbable metabolites of flavonoids can be formed by the colonic microflora, these compounds can have systemic effects on XO. Hence, their effects on XO-catalyzed oxidation of 6-MP and xanthine were also evaluated. In the first screening, these metabolites were tested in a concentration of 20 μM with both 6-MP and xanthine as substrates (both at 5 μM). Most colonic metabolites (3H4MPAA, 24DHAP, HIPA, BA, 24DHBA, 2HPAA, 4HMPAA, 4HBA, 4HPAA, 324DHPPA, 34HPPA, 33HPPA, 3CA, 2H4MBA, 3PPA, and PYR) induced significant decreases of 6-TU formation, while only 2H4MBA and PYR inhibited of uric acid formation significantly (Figure 8). These results demonstrate that most of the colon metabolites are significantly stronger inhibitors of 6-MP than xanthine oxidation. PYR was the only colon metabolite which induced more than 50% inhibitory effect at 20 μM concentration (in both assays). Therefore, the concentration-dependent inhibitory effect of PYR on XO was further investigated. Figure 9 demonstrates the XO-catalyzed 6-TU and uric acid formation in the presence of increasing PYR concentrations. Contrary to other colonic metabolites, PYR proved to be a significantly stronger inhibitor of xanthine than 6-MP oxidation, showing an approximately five-fold lower IC_50_ value for uric acid vs. 6-TU formation (Table 1).

### 2.5. Modeling Studies

**Figure 10:**
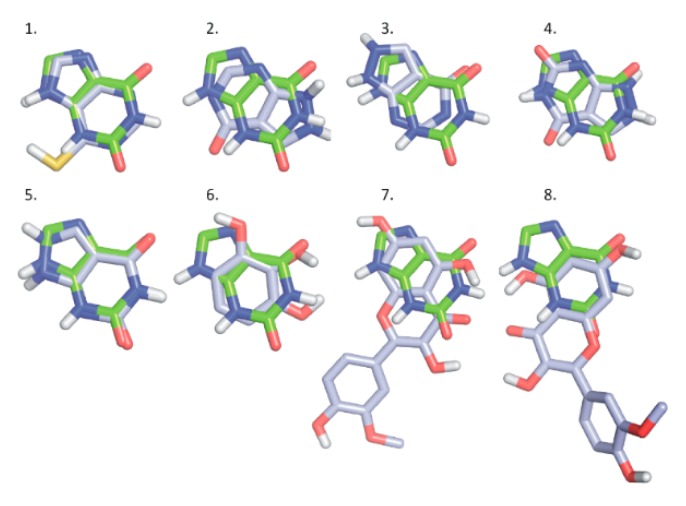
A series of docked ligands (light blue) shown together with the native ligand xanthine (green, used as reference): 6-MP (1; Rank 1); APU (2; Rank 1), APU (3; Rank 2); oxipurinol (4, Rank 1), oxipurinol (5, Rank 2), PYR (6; Rank 1), IR (7; Rank 1), and IR (8, Rank 2).

**Figure 11:**
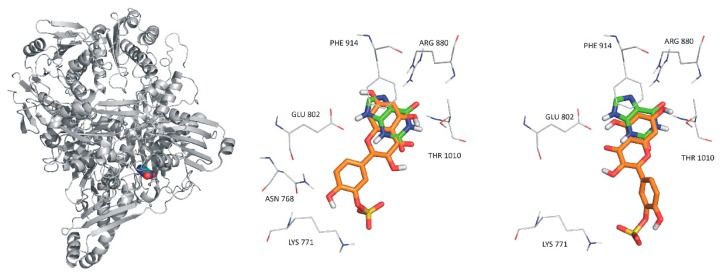
The reference ligand xanthine (spheres, left) occupying its binding site on XO (gray cartoon). In the rest of the panels (middle and right), xanthine is marked with green color. Rank 1 (orange, middle) and Rank 2 (orange, right) binding sites of quercetin-3′sulfate in the XO enzyme.

To test the docking methodology in the present study, the native ligand of XO was removed from the complex PDB structure 3eub, and the ligand-free protein was used for redocking of xanthine. The crystallographic conformation of xanthine (protonation state 1, see in Figure S2) was reproduced at a root mean squared deviation (RMDS) of 1.000 Å where all ten docked conformations found Rank 1 of the lowest ∆Gb (Figure S3). Crystallographic structures are also available for ligands Q [24], 6-MP [25], xanthine [26], and oxipurinol [27] bound to XO (PDB IDs 3nyv, 3ns1, 3eub, 3bdj). The binding pockets of these ligands have the common amino acids surrounding the xanthine pocket Glu802, Ser876, Phe914, Phe1009, and Thr1010 (3eub numbering). Accordingly, the binding positions of the docked ligands Q, IR, Q3′S, 6-MP, xanthine, APU, oxipurinol, and PYR show common pharmacophore regions involving the (2,6)-oxo groups of xanthine (Figure 10). The common target residues were identified in our docking calculations of Q and Q3′S as well (Figure 11). Regarding Q3′S, the sulfate group was involved in a salt bridge with Lys771 which is outside the xanthine pocket and appears as an extra feature of the pharmacophore of this ligand.

## 3. Discussion

Previous studies showed that Q can inhibit XO-catalyzed uric acid formation from xanthine [17,18,28,29]; however, the effects of Q and its metabolites on XO-catalyzed 6-MP oxidation have not been previously reported. Although Q proved to be a strong inhibitor of XO in several in vitro studies, the IC_50_ and α values of Q show large variations. Some reports suggest the stronger [18] while other studies report the weaker [17,28] inhibition of XO-catalyzed uric acid formation by Q compared to APU. A recent study described similar inhibitory potency for Q and APU on xanthine oxidation [29], which is in good agreement with our results. In another study, the inhibitory effects of some sulfate (Q-3-sulfate) and glucuronide (Q3G, Q-7-glucuronide, Q-3′-glucuronide, and Q-4′-glucuronide) conjugates of Q on XO-catalyzed xanthine oxidation were tested [30]. They found that Q3G and Q-7-glucuronide were only weak inhibitors of XO-catalyzed xanthine oxidation, which is consistent with our negative results at low concentrations (0–20 μM) of Q3G. Interestingly, Q-3′-glucuronide and Q-4′-glucuronide were similarly strong inhibitors of xanthine oxidation as Q; however, these conjugates are poorly formed in humans [7,31]. Furthermore, the position of the sulfate group may be also highly relevant because Q-3-sulfate inhibited XO only at very high concentrations [30], while Q3′S proved to be a strong inhibitor in our investigation.

In each enzyme assay (6-MP, xanthine, and hypoxanthine oxidation), APU was applied as a positive control, because oxipurinol decreased the metabolite formation with lower potency (Figure 4 and Figure 7). This observation is in agreement with the previously reported in vitro data [32]. APU itself is also a substrate of XO which can convert it to oxipurinol [14]; therefore, both the parent compound and the metabolite can inhibit the enzyme. Furthermore, during the oxidation of APU, oxipurinol is formed in the active site of XO, and binds strongly to the reduced state of molybdenum in XO [33]. Since the potent interaction of oxipurinol with XO occurs only when molybdenum is presented in its reduced state, and the molybdenum can be reduced by the substrates of XO (such as xanthine or APU), the oxipurinol formed by XO from APU is more effective compared to the simple addition of oxipurinol [33]. This also explains our initially surprising data, since oxipurinol is considered to be the major culprit of XO inhibition. Indeed, the antihyperuricemic effect of APU is attributed mainly to oxipurinol because of its “pseudoirreversible” (very slow dissociation from the enzyme) effect on XO as well as its much longer elimination half-life and peak plasma concentrations vs. APU (due to the rapid formation of oxipurinol by XO or aldehyde oxidase, and its active reabsorption in kidney tubules) [12,33].

Among the Q conjugates tested, methylated (IR, TAM) and sulfated (Q3′S) derivatives exerted strong inhibitory effect on XO enzyme (Table 1). Since both assays were performed with 5 μM substrate concentrations, the IC_50_ values of the tested compounds can be compared. Q, Q3′S, IR, and TAM were similar or more effective inhibitors of xanthine oxidation than APU. However, compared to APU, these metabolites proved to be approximately ten-fold stronger inhibitors of 6-MP oxidation (Table 1). Nevertheless, it is important to note that the observed difference resulted from the considerably weaker inhibition of 6-MP oxidation by APU, while IC_50_ values of Q, Q3′S, IR, and TAM were similar with both substrates.

Furthermore, 6-MP and xanthine at increasing concentrations could still be metabolized in the presence of the inhibitors, showing that the substrates competed with the flavonoid metabolites in both assays and suggesting that the interactions of flavonoids with the XO enzyme were reversible. This hypothesis is also supported by modeling studies because each tested ligand (APU, oxipurinol, Q, Q3′S, and IR) shares the same binding site with xanthine and 6-MP (see in Figure 10 and Figure 11).

Most of the colonic metabolites exerted stronger effect on 6-MP oxidation than xanthine oxidation; only some less potent inhibitors (4MC, 334DHPPA, 34DHBA, 34DHPAA, HVA, and PHLO) showed similar effects in both assays (Figure 8). Interestingly, PYR was the only bacterial metabolite which strongly inhibited the XO-catalyzed 6-MP and xanthine oxidation. Furthermore, PYR was the sole metabolite which behaved similarly to APU, it exerted a significantly stronger inhibitory effect on xanthine than 6-MP oxidation (Figure 9 and Table 1).

It has been hypothesized that flavonoids may be useful in the treatment of hyperuricemia, based on their ability to inhibit the XO-catalyzed uric acid formation in vitro [21]. The results of animal studies are controversial. For example, Q (100 mg/kg) decreased the serum urate levels in hyperuricemic mice significantly [34], whereas in another study, even 400 mg/kg orally administered Q failed to decrease the serum urate levels in either normal or hyperuricemic mice [35]. Interestingly, Zhu et al. [34] reported a considerably stronger effect on serum urate levels in mice after per os than intraperitoneal Q administration, suggesting the potential importance of presystemic metabolite formation. Human studies indicate that Q treatment does not influence the serum uric acid levels at 1000 or 2000 mg/day doses [36,37], and a minimal reduction in serum urate occurs even after a four-week treatment with Q (500 mg/day) [38]. We have found that Q, Q3′S, IR, and TAM have similar inhibitory effects on xanthine oxidation to APU. However, even after repeated daily oral administration of 1000 mg Q, the peak plasma concentrations of total Q are only in the low micromolar range [39], whereas the therapeutic peak plasma concentrations of APU and oxipurinol following a single 200 mg oral dose are ca. 35–40 μM [40]. Furthermore, the effect of Q and Q3′S on XO-catalyzed hypoxanthine oxidation showed that these flavonoids are only three-fold stronger inhibitors of hypoxanthine oxidation vs. APU (Table 2). Therefore, it is very unlikely that the approximately ten-fold lower total Q levels will produce similar therapeutic effects to APU and oxipurinol.

The simultaneous administration of conventional APU and 6-MP (or azathioprine, which is a prodrug of 6-MP) doses can result in toxic consequences, due to the decreased elimination of 6-MP by XO enzyme [16]. Nevertheless, the addition of low dose APU to decreased dose of thiopurine analogues (6-MP or azathioprine) can attenuate the incidence of thiopurine hepatotoxicity. The reason is that APU modulates not only the XO enzyme, but affects the formation of 6-methylmercaptopurine as well [16]. However, careful monitoring for adverse effects is strongly recommended [13]. It has to be again emphasized that APU and oxipurinol reach much higher concentrations in the human circulation (and likely in tissues) than Q and its conjugated metabolites; however, Q and its methyl and sulfate conjugates are approximately ten-fold stronger inhibitors of XO-catalyzed 6-MP oxidation compared to APU (Table 1). Mullen et al. [7] suggest that Q3′S is the dominant circulating metabolite of Q, while Cialdella-Kam et al. [41] describe I3G as the major conjugated metabolite. Q3′S has a three-fold stronger inhibitory effect on 6-MP oxidation than Q (Table 1). The potent interaction of Q3′S with XO may be explained by its interaction with Lys771 outside of the xanthine pocket (Figure 10). Since catechol-O-methyltransferase prefers the 3′-O-methylation of the catechol structure, TAM is only a minor metabolite of Q in humans [42]. However, high I3G levels in the circulation [7,31,41] suggest significant intracellular formation of IR, which is a strong inhibitor of XO-catalyzed 6-MP oxidation (Figure 3). Therefore, it is reasonable to hypothesize that the extremely high intake of Q (e.g., one or more grams daily intake through dietary supplements) may result in the dangerous pharmacokinetic interaction with 6-MP and/or azathioprine [43]. Because some websites suggest that Q exerts anti-inflammatory and antitumor effects, simultaneous administration of Q and thiopurine analogues may occur.

Among the tested microbial metabolites, only PYR strongly inhibited the XO-catalyzed oxidation of 6-MP and xanthine. Considering the fact that sulfate conjugates of PYR reached 10 µM or even higher plasma concentrations after the intake of mixed berry fruit puree [44], PYR-XO interaction may have biological relevance. Furthermore, PYR is apparently a metabolite of more dietary flavonoids and/or polyphenols. Currently, the direct data on plasma concentrations of PYR after high doses of food supplements containing flavonoids are missing; therefore, this hypothesis needs to be confirmed. Nevertheless, the PYR is able to significantly inhibit the XO enzyme; thus, PYR may interfere with uric acid formation and 6-MP oxidation.

## 4. Materials and Methods

### 4.1. Reagents

Quercetin, isorhamnetin, tamarixetin, and 3-coumaric acid were purchased from Extrasynthese. Quercetin-3′-sulfate, quercetin-3-glucuronide, and isorhamnetin-3-glucuronide were synthetized as described [45]. Xanthine oxidase (from bovine milk), allopurinol, 6-mercaptopurine, hypoxanthine, xanthine, uric acid, oxipurinol, 3-phenylpropionic acid, 3-(4-hydroxyphenyl)propionic acid, 3-(2,4-dihydroxyphenyl)propionic acid, 2-hydroxyphenylacetic acid, 4-hydroxyphenylacetic acid, 3,4-dihydroxyphenylacetic acid, 3-hydroxy-4-methoxyphenylacetic acid, 4-(hydroxymethyl)phenylacetic acid, benzoic acid, 4-hydroxybenzoic acid, 2,4-dihydroxybenzoic acid, 3,4-dihydroxybenzoic acid, hippuric acid, 2,4-dihydroxyacetophenon, 4-methylcatechol, resorcinol, pyrogallol, phloroglucinol, 4-methoxysalicylic acid, and homovanillic acid were obtained from Sigma-Aldrich (St. Louis, MO, US). 3-(3-hydroxyphenyl)propionic acid and 3-(3,4-dihydroxyphenyl)propionic acid were purchased from Toronto Research Chemicals. 6-thiouric acid was obtained from Carbosynth, 6-thioxanthine was purchased from 5A Pharmatech. Uric acid was dissolved in 0.01 M sodium hydroxide (2 mM), while Q and its metabolites, APU, 6-MP, 6-TX, 6-TU, and xanthine were dissolved in dimethyl sulfoxide (each 2 mM) and stored at –20 °C.

### 4.2. XO Assay with 6-MP Substrate

The substrate (6-MP; 5 μM) was incubated with 0.01 U/mL XO in the absence and presence of inhibitors (0–20 μM) in a thermomixer (700 rpm, 37 °C; Eppendorf), employing APU and oxipurinol as positive controls. The incubations were carried out in 0.05 M sodium phosphate buffer (pH 7.5; final volume of incubates: 500 μL). The reaction started with the addition of the enzyme, and was stopped with 30 μL of 6 M HClO_4_ 20, 40, or 60 min later. Thereafter, the samples were vortexed and centrifuged at 14,000× *g* for 5 min at room temperature. A 300-μL aliquot of the supernatant was carefully removed, after which 36 μL of 1 M KOH was added to these aliquots. Samples were cooled to 4 °C to enhance the precipitation of the formed KClO_4_, and centrifuged at 14,000× *g* for 5 min at 4 °C. The supernatants were carefully removed and the substrate together with metabolites were analyzed by HPLC (see details in Section 4.5). 6-MP is oxidized to 6-TX followed by 6-TU in the two-step reaction. However, only 6-MP and 6-TU were detectable, likely because 6-TX does not dissociate significantly from the enzyme before the second oxidation step. In the absence of XO enzyme, no metabolite formation was observed.

### 4.3. XO Assay with Xanthine Substrate

Xanthine (5 μM) was incubated with 0.0012 U/mL XO in the absence or presence of inhibitors (0–20 μM). Incubations were started with the addition of the enzyme (final volume of incubates: 500 μL), and stopped after 4, 8, and 12 min with 30 μL of 6 M HClO_4_. Thereafter, the samples were vortexed, after which 97 μL of 1 M KOH was added, then samples were cooled to 4 °C and centrifuged. Other experimental conditions were the same as described in Section 4.2. Xanthine and uric acid were quantified by HPLC (see details in Section 4.5). In the absence of XO, no uric acid formation was detected.

### 4.4. XO Assay with Hypoxanthine Substrate

Hypoxanthine (5 μM) was incubated with 0.012 U/mL XO enzyme in the absence and presence of increasing concentrations of Q, Q3′S, APU or oxipurinol (0.1, 0.5, 1, and 3 μM). The experiments were started with the addition of the enzyme and stopped after 4 min with 30 μL of 6 M HClO_4_. Other experimental conditions were the same as described in Section 4.3. XO-catalyzed oxidation of hypoxanthine results in the formation of xanthine and uric acid in two consecutive steps. During these incubations, both xanthine and uric acid appeared in incubates; therefore, for assaying hypoxanthine oxidation, it was necessary to sum the quantities of xanthine and uric acid formed.

### 4.5. HPLC Analyses

The HPLC system used to determine 6-MP, 6-TX, 6-TU, xanthine, hypoxanthine, and uric acid was equipped with a Rheodyne 7125 injector with a 20-μL sample loop, Waters 510 pump (Milford, MA, USA), and Waters 486 UV-detector. Data were evaluated by Millennium Chromatography Manager Software (Waters).

Quantification of xanthine, hypoxanthine, and uric acid was performed by the previously described method [46] with minor modifications. During the isocratic elution, the mobile phase contained 0.01 M potassium phosphate buffer (pH 4.55) and methanol (98:2 *v*/*v*%). Samples passed through a guard column (Phenomenex Security Guard™ Cartridge C18, 4.0 × 3.0 mm, Torrance, CA, USA) linked to an analytical column (Phenomenex C18, 250 × 4.6 mm, 5 μm) at a 1.1 mL/min flow rate at room temperature. Peak areas were determined at 275 nm. This method was suitable for the separation and quantification of xanthine and uric acid in the presence of most Q metabolites; however, 34DHPAA, 4HMPAA, and PYR co-eluted with xanthine, while 4HBA co-eluted with uric acid. Therefore, for the analysis of xanthine and uric acid in the presence of these metabolites, the mobile phase was modified to 0.01 M potassium phosphate buffer (pH 4.55) and methanol (99:1 *v*/*v*%); the other chromatographic parameters remained unchanged.

Quantitation of 6-MP, 6-TX, and 6-TU was performed by the previously described method [47] with minor modifications. During the isocratic elution, the samples passed through a guard column (Phenomenex Security Guard™ Cartridge C18, 4.0 × 3.0 mm) linked to an analytical column (Phenomenex Gemini® NX-C18, 150 × 4.6 mm, 3 μm), using methanol, acetonitrile, and 0.02 M phosphoric acid (4:5:91 *v*/*v*%) as mobile phase at a 0.8 mL/min flow rate at room temperature. Peak areas were determined at 334 nm.

### 4.6. Modeling Studies

Docking calculations were performed using the AutoDock 4.2 program package (Molecular Graphics Laboratory, Department of Molecular Biology, The Scripps Research Institute La Jolla, San Diego, CA, USA). Ligand structures of Q, IR, Q3′S, 6-MP, xanthine, APU, oxipurinol, and PYR were obtained from PubChem (National Center for Biotechnology Information, US National Library of Medicine, Bethesda, MD, USA) [48] as Spatial Data File, and hydrogenated using Avogadro [49]. Regarding the two protonation states of xanthine, the first one was set (Figure S2).

Energy-minimization of each ligand molecule was performed by the semiempirical quantum chemistry program package MOPAC (ver. 17.279L) (Stewart Computational Chemistry, Colorado Springs, CO, US) [50]. The geometries were optimized at a 0.001 gradient norm and subjected to subsequent force calculations using PM7 parameterization. In each calculation, the force constant matrices were positive definite. The structure of XO (PDB code 3eub) was used as a target of docking without the native ligand. Gasteiger-Marsilli partial charges [51] were added to the ligands as calculated by AutoDock 4.2 Tools [52].

During target preparation, the protein part was separated from the non-amino acid residues. For the protein part, a two-step energy minimization was performed. A steepest descent minimization was followed by a conjugate gradient run using convergence thresholds of 1000 and 10 kJ × mol × nm-1, respectively. Non-amino acid residues hydroxy(dioxo)molybdenum and phosphonic acid mono-(2-amino-5,6-dimercapto-4-oxo-3,7,8a,9,10,10a-hexahydro-4h-8-oxa-1,3,9,10-tetraaza-anthracen-7-ylmethyl)ester were prepared as the ligand molecules as described above. During MOPAC energy-minimization, their heavy atoms were position restrained. Finally, the target was reconstructed by merging the prepared protein parts with the non-amino acid residues.

A Kollman united atom representation was applied for target groups with non-polar bonds. Regarding focused docking, the grid box was centered on the center of xanthine in 3eub. A grid map was calculated by AutoGrid 4 [52] with a box size of 60 × 60 × 60 points and 0.375 Å spacing. In each calculation, the number of LGA docking runs was set to 10, numbers of energy evaluations and generations were 20 million. Ligand conformations resulted from the docking runs were ordered by the corresponding calculated binding free energy (∆Gb) values and clustered using a tolerance of 1.75 Å distance between cluster members. Conformations with the lowest ∆Gb within a cluster were selected as cluster representatives and discussed in the text.

### 4.7. Statistical Analyses

Data represent mean ± SEM values derived from at least three independent experiments. Statistical analyses were performed employing one-way ANOVA and Student’s *t*-test (IBM SPSS Statistics, Version 21, Armonk, NY, USA). The minimal level of significance was set to *p* < 0.05.

## Figures and Tables

**Table 1:** Inhibition of XO-catalyzed 6-TU formation and uric acid formation by APU, Q, Q3′S, IR, TAM, Q3G, I3G, and PYR. IC_50_: concentration of the compound which induces 50% inhibition of metabolite formation, IC_50(rel)_ = IC_50_ of the inhibitor divided by the substrate concentration (5 μM 6-MP), α = IC_50_ of the inhibitor divided by IC_50_ of the positive control.

Test Compound	6-MP Oxidation			Xanthine Oxidation			IC_50_ (6-MP)/IC_50_ (Xanthine)
	IC_50_ (μM)	IC_50(rel)_	α	IC_50_ (μM)	IC_50(rel)_	α	
APU (positive ctrl)	7.00	1.40	1.00	0.60	0.12	1.00	11.67
Q	1.40	0.28	0.20	0.80	0.16	1.33	1.75
Q3′S	0.50	0.10	0.07	0.40	0.08	0.67	1.25
IR	0.60	0.12	0.09	0.70	0.14	1.17	0.86
TAM	0.20	0.04	0.03	0.20	0.04	0.33	1.00
Q3G	>20.0	>4.0	-	>20.0	>4.0	-	-
I3G	>20.0	>4.0	-	>20.0	>4.0	-	-
PYR	10.10	2.02	1.44	1.80	0.36	3.00	5.61

**Table 2:** Inhibition of XO-catalyzed hypoxanthine oxidation by Q, Q3′S, APU, and oxipurinol. IC_50_: concentration of the compound which induces 50% inhibition of metabolite formation, IC_50(rel)_ = IC_50_ of the inhibitor divided by the substrate concentration (5 μM hypoxanthine), α = IC_50_ of the inhibitor divided by IC_50_ of the positive control (APU).

Test Compound	IC_50_ (μM)	IC_50(rel)_	α
APU (positive ctrl)	0.66	0.13	1.00
Q	0.24	0.05	0.36
Q3′S	0.21	0.04	0.32
Oxipurinol	>3.00	>0.60	-

## Supplementary Materials

Supplementary materials can be found at https://www.mdpi.com/1422-0067/20/11/2681/s1.

## Abbreviations

24DHAP	2,4-Dihydroxyacetophenon
24DHBA	2,4-Dihydroxybenzoic acid
2H4MBA	4-Methoxysalicylic acid
2HPAA	2-Hydroxyphenylacetic acid
324DHPPA	3-(2,4-Dihydroxyphenyl)propionic acid
334DHPPA	3-(3,4-Dihydroxyphenyl)propionic acid
33HPPA	3-(3-Hydroxyphenyl)propionic acid
34DHBA	3,4-Dihydroxybenzoic acid
34DHPAA	3,4-Dihydroxyphenylacetic acid
34HPPA	3-(4-Hydroxyphenyl)propionic acid
3CA	3-Coumaric acid
3H4MPAA	3-Hydroxy-4-methoxyphenylacetic acid
3PPA	3-Phenylpropionic acid
4HBA	4-Hydroxybenzoic acid
4HMPAA	4-(Hydroxymethyl)phenylacetic acid
4MC	4-Methylcatechol
6-MP	6-Mercaptopurine
6-TU	6-Thiouric acid
6-TX	6-Thioxanthine
APU	Allopurinol
BA	Benzoic acid
HIPA	Hippuric acid
HVA	Homovanillic acid
I3G	Isorhamnetin-3-glucuronide
IR	Isorhamnetin
PHLO	Phloroglucinol
PYR	Pyrogallol
Q	Quercetin
Q3G	Quercetin-3-glucuronide
Q3′S	Quercetin-3′-sulfate
RES	Resorcinol
TAM	Tamarixetin
XO	Xanthine oxidase

## References

[1] Formica J.V., Regelson W. (1995). Review of the biology of Quercetin and related bioflavonoids. Food Chem. Toxicol..

[2] Hollman P.C., De Vries J.H., Van Leeuwen S.D., Mengellers M.J., Katan M.B. (1995). Absorption of dietary quercetin glycosides and quercetin in healthy ileostomy volunteers. Am. J. Clin. Nutr..

[3] Kelly G.S. (2011). Quercetin. Altern. Med. Rev..

[4] Manach C., Texier O., Regerat F., Agullo G., Demigne C., Remesy C. (1996). Dietary quercetin is recovered in rat plasma as conjugated derivatives of isorhamnetin and quercetin. J. Nutr. Biochem..

[5] Terao J., Murota K., Kawai Y. (2011). Conjugated quercetin glucuronides as bioactive metabolites and precursors of aglycone in vivo. Food Funct..

[6] Del Rio D., Rodriguez-Mateos A., Spencer J.P.E., Tognolini M., Borges G., Crozier A. (2013). Dietary (poly)phenolics in human health: Structures, bioavailability, and evidence of protective effects against chronic diseases. Antioxid. Redox Signal..

[7] Mullen W., Edwards C.A., Crozier A. (2006). Absorption, excretion and metabolite profiling of methyl-, glucuronyl-, glucosyl- and sulpho-conjugates of quercetin in human plasma and urine after ingestion of onions. Br. J. Nutr..

[8] Conquer J.A., Maiani G., Azzini E., Raguzzini A., Holub B.J. (1998). Supplementation with quercetin markedly increases plasma quercetin concentration without effect on selected risk factors for heart disease in healthy subjects. J. Nutr..

[9] Rechner A.R., Kuhnle G., Bremner P., Hubbard G.P., Moore K.P., Rice-Evans C.A. (2002). The metabolic fate of dietary polyphenols in humans. Free Rad. Biol. Med..

[10] Aura A.M. (2008). Microbial metabolism of dietary phenolic compounds in the colon. Phytochem. Rev..

[11] Serra A., Macia A., Romero M.P., Reguant J., Ortega N., Motilva M.J. (2012). Metabolic pathways of the colonic metabolism of flavonoids (flavonols, flavones and flavanones) and phenolic acids. Food Chem..

[12] Day R.O., Graham G.G., Hicks M., McLachlan A.J., Stocker S.L., Williams K.M. (2007). Clinical pharmacokinetics and pharmacodynamics of allopurinol and oxypurinol. Clin. Pharmacokinet..

[13] Leong R.W., Gearry R.B., Sparrow M.P. (2008). Thiopurine hepatotoxicity in inflammatory bowel disease: The role for adding allopurinol. Expert Opin. Drug Saf..

[14] Galbusera C., Orth P., Fedida D., Spector T. (2006). Superoxide radical production by allopurinol and xanthine oxidase. Biochem. Pharmacol..

[15] Berry C.E., Hare J.M. (2004). Xanthine oxidoreductase and cardiovascular disease: Molecular mechanisms and pathophysiological implications. J. Physiol..

[16] McLeod H.L. (1998). Clinically relevant drug-drug interactions in oncology. Br. J. Clin. Pharmacol..

[17] Lin C.M., Chen C.S., Chen C.T., Liang Y.C., Lin J.K. (2002). Molecular modeling of flavonoids that inhibits xanthine oxidase. Biochem. Biophys. Res. Commun..

[18] Van Hoorn D.E.C., Nijveldt R.J., Van Leeuwen P.A.M., Hofman Z., M’Rabet L., De Bont D.B.A., Van Norren K. (2002). Accurate prediction of xanthine oxidase inhibition based on the structure of flavonoids. Eur. J. Pharmacol..

[19] Mladenka P., Zatloukalová L., Filipský T., Hrdina R. (2010). Cardiovascular effects of flavonoids are not caused only by direct antioxidant activity. Free Radic. Biol. Med..

[20] Iio M., Moriyama A., Matsumoto Y., Takaki N., Fukumoto M. (1985). Inhibition of Xanthine Oxidase by Flavonoids. Agric. Biol. Chem..

[21] Nagao A., Seki M., Kobayashi H. (1999). Inhibition of xanthine oxidase by flavonoids. Biosci. Biotechnol. Biochem..

[22] Miron A., Aprotosoaie A.C., Trifan A., Xiao J. (2017). Flavonoids as modulators of metabolic enzymes and drug transporters. Ann. N. Y. Acad. Sci..

[23] Poór M., Boda G., Needs P.W., Kroon P.A., Lemli B., Bencsik T. (2017). Interaction of quercetin and its metabolites with warfarin: Displacement of warfarin from serum albumin and inhibition of CYP2C9 enzyme. Biomed. Pharmacother..

[24] Cao H., Pauff J.M., Hille R. (2014). X-ray crystal structure of a xanthine oxidase complex with the flavonoid inhibitor quercetin. J. Nat. Prod..

[25] Cao H., Pauff J.M., Hille R. (2010). Substrate orientation and catalytic specificity in the action of xanthine oxidase: The sequential hydroxylation of hypoxanthine to uric acid. J. Biol. Chem..

[26] Pauff J.M., Cao H., Hille R. (2009). Substrate Orientation and Catalysis at the Molybdenum Site in Xanthine Oxidase: CRYSTAL STRUCTURES IN COMPLEX WITH XANTHINE AND LUMAZINE. J. Biol. Chem..

[27] Okamoto K., Eger B.T., Nishino T., Pai E.F., Nishino T. (2008). Mechanism of inhibition of xanthine oxidoreductase by allopurinol: Crystal structure of reduced bovine milk xanthine oxidoreductase bound with oxipurinol. Nucleosides Nucleotides Nucleic Acids.

[28] Cos P., Ying L., Calomne M., Hu J.P., Cimanga K., Van Poel B., Pieters L., Vlietinck A.J., Berghe D.V. (1998). Structure-activity relationship and classification of flavonoids as inhibitors of xanthine oxidase and superoxide scavengers. J. Nat. Prod..

[29] Zhang C., Wang R., Zhang G., Gong D. (2018). Mechanistic insights into the inhibition of quercetin on xanthine oxidase. Int. J. Biol. Macromol..

[30] Day A.J., Bao Y., Morgan M.R., Williamson G. (2000). Conjugation position of quercetin glucuronides and effect on biological activity. Free Radic. Biol. Med..

[31] Day A.J., Mellon F., Barron D., Sarrazin G., Morgan M.R., Williamson G. (2001). Human metabolism of dietary flavonoids: Identification of plasma metabolites of quercetin. Free Radic. Res..

[32] Elion G.B. (1966). Enzymatic and metabolic studies with allopurinol. Ann. Rheum. Dis..

[33] Spector T. (1977). Inhibition of urate production by allopurinol. Biochem. Pharmacol..

[34] Zhu J.X., Wang Y., Kong L.D., Yang C., Zhang X. (2004). Effects of Biota orientalis extract and its flavonoid constituents, quercetin and rutin on serum uric acid levels in oxonate-induced mice and xanthine dehydrogenase and xanthine oxidase activities in mouse liver. J. Ethnopharmacol..

[35] Huang J., Wang S., Zhu M., Chen J., Zhu X. (2011). Effects of Genistein, Apigenin, Quercetin, Rutin and Astilbin on serum uric acid levels and xanthine oxidase activities in normal and hyperuricemic mice. Food Chem. Toxicol..

[36] Abbey E.L., Rankin J.W. (2011). Effect of quercetin supplementation on repeated-sprint performance, xanthine oxidase activity, and inflammation. Int. J. Sport Nutr. Exerc. Metab..

[37] Boots A.W., Drent M., De Boer V.C., Bast A., Haenen G.R. (2011). Quercetin reduces markers of oxidative stress and inflammation in sarcoidosis. Clin. Nutr..

[38] Shi Y., Williamson G. (2016). Quercetin lowers plasma uric acid in pre-hyperuricaemic males: A randomised, double-blinded, placebo-controlled, cross-over trial. Br. J. Nutr..

[39] Heinz S.A., Henson D.A., Nieman D.C., Austin M.D. (2010). A 12-week supplementation with quercetin does not affect natural killer cell activity, granulocyte oxidative burst activity or granulocyte phagocytosis in female human subjects. Br. J. Nutr..

[40] Turnheim K., Krivanek P., Oberbauer R. (1999). Pharmacokinetics and pharmacodynamics of allopurinol in elderly and young subjects. Br. J. Clin. Pharmacol..

[41] Cialdella-Kam L., Nieman D.C., Sha W., Meaney M.P., Knab A.M., Shanely R.A. (2013). Dose-response to 3 months of quercetin-containing supplements on metabolite and quercetin conjugate profile in adults. Br. J. Nutr..

[42] De Santi C., Pietrabissa A., Mosca F., Pacifici G.M. (2002). Methylation of quercetin and fisetin, flavonoids widely distributed in edible vegetables, fruits and wine, by human liver. Int. J. Clin. Pharmacol. Ther..

[43] Vida R.G., Fittler A., Somogyi-Végh A., Poór M. (2019). Dietary quercetin supplements: Assessment of online product informations and quantitation of quercetin in the products by high performance liquid chromatography. Phytother. Res..

[44] Pimpão R.C., Ventura M.R., Ferreira R.B., Williamson G., Santos C.N. (2015). Phenolic sulfates as new and highly abundant metabolites in human plasma after ingestion of a mixed berry fruit purée. Br. J. Nutr..

[45] Needs P.W., Kroon P.A. (2006). Convenient synthesis of metabolically important glucuronides and sulfates. Tetrahedron.

[46] Mei D.A., Gross G.J., Nithipatikom K. (1996). Simultaneous determination of adenosine, inosine, hypoxanthine, xanthine, and uric acid in microdialysis samples using microbore column high-performance liquid chromatography with a diode array detector. Anal. Biochem..

[47] Hawwa A.F., Millership J.S., Collier P.S., McElnay J.C. (2009). Development and validation of an HPLC method for the rapid and simultaneous determination of 6-mercaptopurine and four of its metabolites in plasma and red blood cells. J. Pharm. Biomed. Anal..

[48] Kim S., Thiessen P.A., Bolton E.E., Chen J., Fu G., Gindulyte A., Han L., He J., He S., Shoemaker B.A., Wang J. (2016). PubChem Substance and Compound databases. Nucleic Acids Res..

[49] Hanwell M.D., Curtis D.E., Lonie D.C., Vandermeersch T., Zurek E., Hutchison G.R. (2012). Avogadro: An advanced semantic chemical editor, visualization, and analysis platform. J. Cheminform..

[50] Stewart J.J.P. (1990). MOPAC: A semiempirical molecular orbital program. Computer-Aided Mol. Des..

[51] Gasteiger J., Marsili M. (1980). Iterative partial equalization of orbital electronegativity—a rapid access to atomic charges. Tetrahedron.

[52] Morris G.M., Huey R., Lindstrom W., Sanner M.F., Belew R.K., Goodsell D.S., Olson A.J. (2009). AutoDock4 and AutoDockTools4: Automated docking with selective receptor flexibility. J. Com. Chem..

