# Supplementary material for: Inhibitory Effects of Quercetin and Its Human and Microbial Metabolites on Xanthine Oxidase Enzyme
Source: Int J Mol Sci. 2019 May 31;20(11):2681. doi: 10.3390/ijms20112681 (PMC6600370; doi:10.3390/ijms20112681)
Supplement: Supplementary file 1 [file ijms-20-02681-s001.pdf]

International Journal of *Molecular Sciences*

MDPI

Supplementary Materials: Inhibitory Effects of Quercetin and Its Human and Microbial Metabolites on Xanthine Oxidase Enzyme

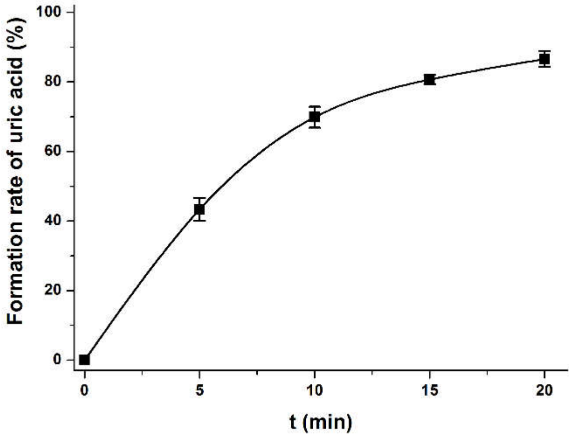

Figure S1. The time course of XO-catalyzed formation of uric acid from xanthine (see details in the Materials and Methods). Graph depicts % conversion of xanthine to uric acid in the absence of inhibitors.

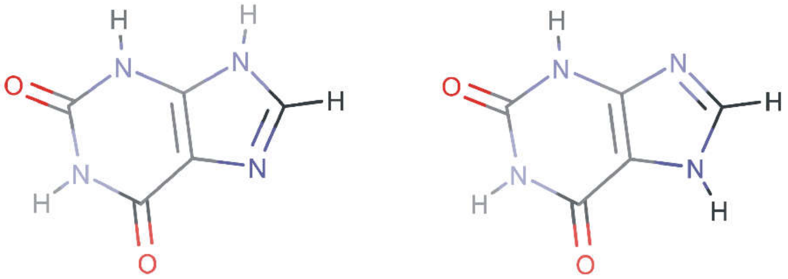

Figure S2. Protonation states one (left) and two (right) of xanthine.

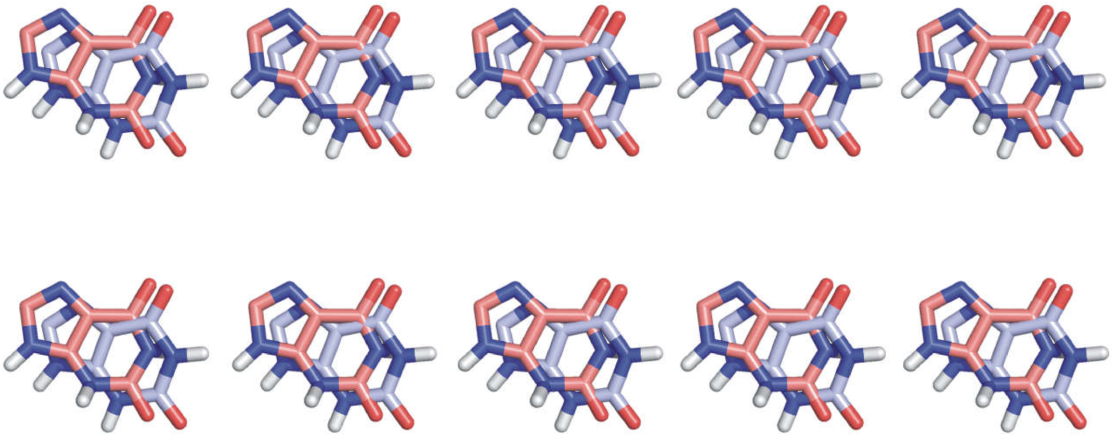

Figure S3. All ten docked ligand conformations (light blue) shown together with the ligand xanthine from 3eub (red, used as reference).
